# Luminescence Properties of ZrO_2_: Ti Ceramics Irradiated with Electrons and High-Energy Xe Ions

**DOI:** 10.3390/ma17061307

**Published:** 2024-03-12

**Authors:** Alma Dauletbekova, Sergey Zvonarev, Sergey Nikiforov, Abdirash Akilbekov, Tatiana Shtang, Natalia Karavannova, Aiman Akylbekova, Alexey Ishchenko, Gulzhanat Akhmetova-Abdik, Zein Baymukhanov, Gulnara Aralbayeva, Guldar Baubekova, Anatoli I. Popov

**Affiliations:** 1 Department of Technical Physics, L.N. Gumilyov Eurasian National University, Satpayev Str. 2, Astana 010008, Kazakhstan; akilbekov_at@enu.kz (A.A.); aiman88_88@mail.ru (A.A.); gulzhanatakhmet@gmail.com (G.A.-A.); zeinb77@mail.ru (Z.B.); agm_555@mail.ru (G.A.); guldar_87@mail.ru (G.B.); 2 Institute of Physics and Technology, Ural Federal University, 19 Mira str., Yekaterinburg 620002, Russia; s.v.zvonarev@urfu.ru (S.Z.); s.v.nikiforov@urfu.ru (S.N.); t.v.shtang@urfu.ru (T.S.); natalia.karavannova@urfu.me (N.K.); a.v.ishchenko@urfu.ru (A.I.); 3 Institute of Solid State Physics, University of Latvia, Kengaraga str. 8, LV-1063 Riga, Latvia; anatoli.popov@cfi.lu.lv

**Keywords:** zirconium dioxide, thermoluminescence, pulse cathodoluminescence, ceramics, scanning electron microscopy, high-energy ions, electron beam

## Abstract

Samples of ZrO_2_ ceramics with different concentrations of impurity titanium ions were synthesized by mixing zirconium and titanium oxide powders in different mass ratios. The X-ray diffraction analysis was used to determine the phase composition, lattice parameters, and crystallite size of the ceramics with varying dopant concentrations. Upon irradiation of the samples with 220 MeV Xe ions to a fluence of 10^10^ ions/cm^2^, a decrease in the intensity of the pulsed cathodoluminescence band at 2.5 eV was observed. Additionally, ion irradiation resulted in the emergence of a new thermoluminescence peak at 450–650 K attributed to radiation-induced traps of charge carriers. Further analysis revealed that the thermoluminescence curves of samples irradiated with electrons and ions comprise a superposition of several elementary peaks. Notably, a complex non-monotonic dependence of cathodo- and thermoluminescence intensity on titanium concentration was observed, suggesting the influence of concentration quenching and the presence of tunneling transitions.

## 1. Introduction

Zirconium dioxide (ZrO_2_) stands out for its high refractive index, and thermal and chemical stability, making it a versatile material used across various industries. It serves diverse purposes, including use in heat-shielding coatings, electron-optical and biochemical materials, and ionizing radiation detectors [1,2,3]. Among the different types of detectors, luminescent detectors and thermoluminescent (TL) dosimeters hold particular significance. Thermoluminescence dosimetry (TLD) involves the use of crystals or ceramics with specific lattice defects of impurity and/or intrinsic origin, known as crystal phosphors. These substances exhibit the ability to emit light when exposed to ionizing radiation and are subsequently heated. In this process, the quantity of ionizing radiation absorbed is directly proportional to the number of emitted quanta [4]. TL dosimetry is widely used for the individual dosimetry of personnel, environmental monitoring, and medical exposure control. A wide spectrum of materials is employed in thermoluminescent detectors, showcasing the versatility of the technique. Examples include alkaline-halides (e.g., LiF: Mg, Ti; LiF: Mg, Cu, P), sulphates (e.g., BaSO_4_: Eu, CaSO_4_: Dy), borates (e.g., MgB_4_O_7_: Dy), oxides (e.g., Al_2_O_3_, ZnO, ZrO_2_, MgO), and complex compounds (e.g., K_3_Na(SO_4_)_2_: Eu, Ba_0,97_Ca_0,03_SO_4_: Eu, YAG etc.) [5,6,7,8,9,10,11,12,13,14,15,16,17,18,19,20,21,22,23,24,25,26,27,28,29,30].

It is established that the luminescent properties of nominally pure and doped zirconium dioxide (ZrO_2_) rely on the preparation method, grain size, crystal structure and sample morphology [31,32,33,34,35,36,37,38,39,40,41,42]. Nominally, pure ZrO_2_ is characterized by its own luminescence at 2.5–2.7 eV (470–490 nm) [31,32,37,39]. According to some perspectives, the indicated luminescence band in ZrO_2_ is due to the relaxation of centers associated with intrinsic defects in the anionic sublattice (such as oxygen vacancies) [32,33,37]. An alternative viewpoint associates the origin of the luminescence band at 2.6 eV with the relaxation of impurity ions of titanium [34,36,38]. According to the literature [31,36], raw ZrO_2_ inevitably contains traces of TiO_2_ and this cannot be avoided in any Zr-containing raw materials.

The introduction of titanium as a dopant can significantly change the luminescent properties of zirconium dioxide. For instance, doping nanostructured ZrO_2_ samples with titanium (0.5 mol%) led to an increase in photoluminescence (PL) at 480 nm, under UV radiation excitation [38]. Other researchers have also observed a similar trend of increasing dopant concentration correlating with enhanced intensity of the aforementioned PL band. In this case, the maximum intensity was achieved at a titanium concentration of 0.15 weight % [34]. However, at higher concentrations, a decrease in PL was recorded. Similarly, an analogous increase in TL intensity was observed upon doping ZrO_2_ with titanium (0.1 mol%) [43,44].

The study of the role of titanium impurities in shaping the luminescent properties of zirconium dioxide under irradiation with high-energy ions, particularly heavy xenon ions exceeding 1 MeV, holds significant interest. It is well-established that under such irradiation, new radiation-induced defects and their complexes form in oxide dielectrics [45,46]. These complexes can include both oxygen vacancies and impurity ions in different charge states. Previous research [47] has indicated that complex defects containing oxygen vacancies and titanium ions could be responsible for the formation of a luminescence band at 2.6 eV. In this study, these defects were induced through high-temperature annealing of nominally pure nanostructured zirconium dioxide compacts in a vacuum under reducing conditions provided by the presence of carbon in the graphite form. It is conceivable that such vacancy-impurity complexes could also be generated in samples pre-doped with titanium as a result of high-intense ion irradiation. In this case, elucidating the influence patterns of the introduced titanium impurity concentration on the luminescence intensity of irradiated samples becomes particularly intriguing. Additionally, it is pertinent to investigate changes in luminescent properties for ZrO_2_:Ti ceramics under irradiation with low-energy electrons (130 keV) for comparative analysis. Unlike ion radiation, electronic radiation does not lead to the formation of new radiation-induced defects in the material under study; rather, it changes the charge state of existing ones. In practical terms, the outcomes of this study hold utility for the advancement of luminescent detectors catering to both ion and electron radiation.

It is known that high-energy ion beams (over 1 MeV) are extensively utilized in medicine, industry, and science for applications such as sterilizing food products, purifying wastewater from organic contaminants, oil products, and textile industry wastes, and for the radiation modification of properties in composite materials as well as metals and alloys. High-dose (over 1 kGy) electron radiation with energies of 100–300 keV is employed for surface sterilization and also for the spectroscopy of intrinsic and impurity defects in semiconductor and dielectric materials. From a practical perspective, the results of this work will be useful in developing thermoluminescent detectors for ion and electron radiation based on synthesized ceramics [48].

The purpose of this study is to synthesize samples of ZrO_2_ ceramics with varying dopant concentrations and assess the impact of titanium impurities on the luminescent properties of samples irradiated with xenon ions and electrons.

## 2. Materials and Methods

The samples under investigation were obtained by mixing zirconium and titanium dioxides in the following ratios (ZrO_2_:TiO_2_): 99.9:0.1; 99:1; 95:5; 90:10, and 85:15 wt.%. A 99.1% purity zirconium dioxide nanopowder with a particle size in the range of 30–70 nm was used as the starting material, obtained by the plasma chemical method (Plasmotherm Company, Moscow, Russia), and titanium dioxide powder (150–200 nm, purity 99.8%, Component Reagent PRO company, Saint-Petersburg, Russia). The powders were blended in their as-received state via the dry method using an agate mortar with ethyl alcohol as a binder. The mixing duration was 1–2 h for a 2 g powder quantity, ensuring optimal homogenization to achieve a dry consistency. This mixing time was determined based on the chemical process duration required for the transition from a liquid state of the powder mixture with ethyl alcohol to a dry consistency and was chosen empirically. Samples in the form of disks were obtained from a mixture of powders (100 mg for each compound) by cold uniaxial pressing at a pressure of 500 MPa. The diameter of the resulting compacts was 6 mm, and the thickness was 1–1.5 mm. The compressed pellets were annealed in air in a high-temperature furnace (Linn High Therm HT-1800-M) at a temperature of 1200 °C for 1 h. The selected mode ensured the mechanical strength of the samples. Additionally, in this case, the formation of the ZrTiO_4_ phase did not occur, which would have led to a sharp decrease in luminescence intensity. The chosen method facilitated the production of ZrO_2_:Ti ceramics samples with different dopant concentrations.

The luminescent properties of ceramics were analyzed using pulsed cathodoluminescence (PCL) and TL methods. For TL excitation, the studied samples were irradiated with xenon ions with an energy of 220 MeV (at a fluence of 10^10^ ions/cm^2^) at the DC-60 heavy ion accelerator (Astana, Republic of Kazakhstan). To facilitate a comparative analysis of luminescent properties, the samples were also irradiated with a pulsed electron beam (60 A/cm^2^, 2 ns) from the RADAN EXPERT accelerator with an electron energy of 130 keV at room temperature. The electron irradiation dose was 1.5 kGy per pulse. The dose of radiation delivered by a single pulse of the electron beam at the sample location was determined using a film dosimeter SO PD(F)R-5/50, considering the pulse duration (2 ns), resulting in a dose rate of 1.25∙10^7^ kGy/h. Additionally, the same electron beam was utilized for PCL excitation. The measurement of thermoluminescence (TL) curves was conducted using the standard procedure adopted for commercial TL readers. The sample was placed on a heating element made of a nickel foil plate. During this process, the samples were heated to a predetermined final temperature at a constant rate of 2 K/s. The control of heating and the measurement of the sample’s temperature were facilitated by a special electronic module of the thermostat and a chromel–alumel thermocouple. Throughout the heating process, a photoelectron multiplier periodically measured the photocurrent, which is proportional to the TL intensity. To register the thermoluminescence, an FEU-130 photomultiplier tube with a maximum spectral sensitivity of 400–420 nm was used.

X-ray phase analysis of the samples was conducted using the XRD-7000 X-ray diffractometer (Shimadzu, Japan), equipped with an X-ray tube with a copper anode. Quantitative phase analysis was performed using the full-profile Rietveld method with the X’Pert HighScore Plus software 4.0 (PANalytical, The Netherlands).

To study the morphology of the samples, a ZEISS SIGMA VP scanning electron microscope with an internal secondary electron detector (In-lense), (Jena, Germany), was used in a high-vacuum mode at an accelerating voltage of 2–3.9 kV.

## 3. Results and Discussion

**Figure 1:**
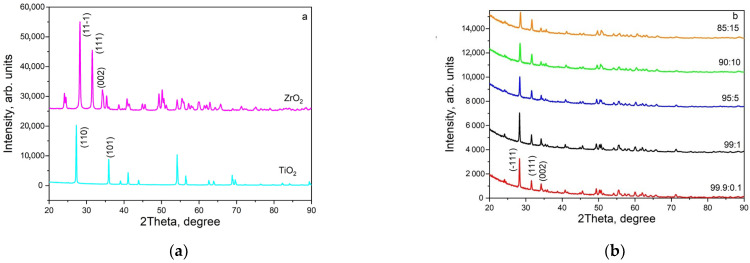
X-ray diffractograms of the original ZrO_2_ and TiO_2_ powders (**a**) and synthesized ceramics with various concentrations of introduced TiO_2_ (**b**).

The phase composition and crystal structure of the ceramic samples were determined by X-ray diffraction (XRD). Phase identification was conducted using an ICDD PDF-2 database (JCPDS 01-083-0944 for monoclinic ZrO_2_ and JCPDS 01-077-0442 for rutile). The obtained diffractograms are shown in Figure 1.

**Figure 2:**
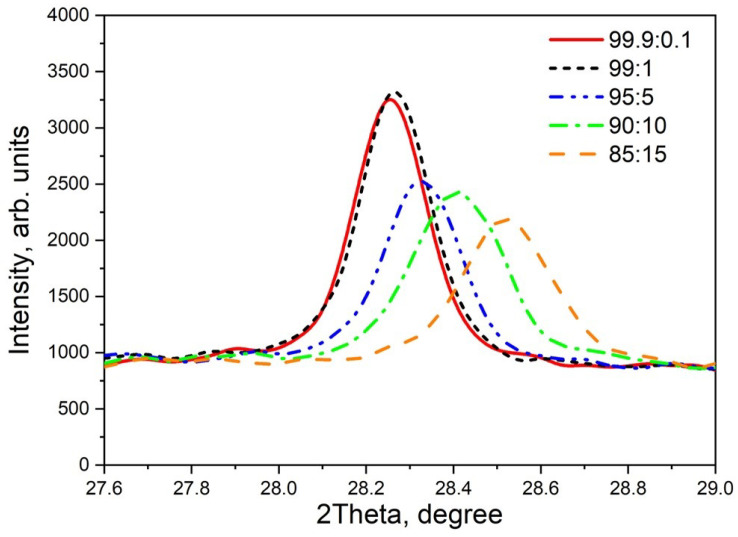
Dependence of the maximum position of the diffraction reflex 2θ = 28.3° on the dopant concentration.

The analysis of Figure 1a shows that the original powders used for synthesis are single-phase systems (monoclinic zirconium dioxide (baddeleyite) and tetragonal TiO_2_ (rutile)). The figure indicates the most characteristic reflexes for the mentioned phases. In ceramics with the selected synthesis parameters, only one phase of monoclinic ZrO_2_ is observed (Figure 1b). The rutile phase was absent in the synthesized ceramics, indicating the incorporation of titanium ions into the ZrO_2_ lattice. No discernible differences in phase composition were detected across varying titanium dioxide concentrations. The diffraction reflexes’ maxima positions shift as the dopant content varies (Figure 2). This effect is shown in more detail in Figure 2 for the reflex at 2θ = 28.3°, which corresponds to the crystallographic plane (−1 1 1). It can be observed that when the concentration of injected TiO_2_ is 5% or higher, the peak position of the reflex shifts towards larger values of 2θ. Moreover, as the dopant concentration increases, this shift becomes more pronounced. These results indicate changes in lattice parameters when doped with varying concentrations of titanium. The lattice parameters of ceramics with varying titanium impurity content, as determined from X-ray diffraction data, are listed in Table 1.

As the titanium concentration increases, the monoclinic lattice parameters a and b decrease monotonically, indicating the replacement of zirconium ions within the lattice. This result aligns with the radius ratio of Zr^4+^ and Ti^4+^ ions, which are 0.79 Å and 0.68 Å, respectively [36]. Using the Scherrer method with XRD data, we calculated the size of crystallites in the ceramics, assuming the particles were spherically symmetric. The half-width values of the most intense reflex at 28.3° corresponding to Miller indices ($\bar{1}$ 1 1) were used for the calculation. The obtained results are shown in Table 1. As the concentration of introduced TiO_2_ increases, the diffraction peak broadens, indicating a decrease in the average grain size from 200 to 80 nm. A similar change in crystallite size has been previously observed with increasing alloying impurity content in other materials such as PbS:Sr and PbS:Fe [49,50]. A slight increase in crystallite size of the doped samples (80–200 nm) compared to the original ZrO_2_ nanopowder (30–70 nm) could be attributed to the heat treatment during synthesis (T = 1200 °C). It is known that the heat treatment of nanopowders leads to a recrystallization effect and grain size growth [51]. It has been observed that exposing oxide materials to high-energy particles, especially fast electrons, can alter their crystal structure [52]. Our research indicates that when ZrO_2_:Ti ceramics were subjected to electron irradiation (130 keV, 15 kGy) and ion irradiation (220 MeV, fluence of 10^10^ ions/cm^2^), no significant changes were observed in the X-ray diffractograms. However, it is possible that at higher irradiation fluences, the effect of irradiation on XRD measurements may become apparent, necessitating further studies.

**Figure 3:**
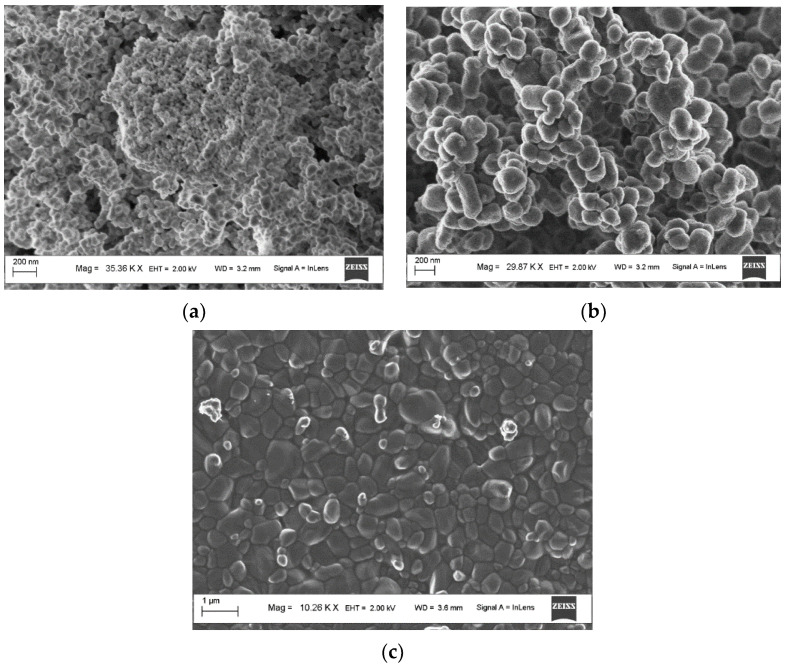
SEM images of the original powders of zirconium dioxide (**a**) and titanium (**b**), as well as ZrO_2_:Ti ceramics with an introduced TiO_2_ concentration of 5% (**c**).

Figure 3 presents SEM images of the original powders of zirconium and titanium dioxides, as well as ZrO_2_:Ti ceramics with an introduced TiO_2_ concentration of 5%. The results show that the crystallite size in the original powders generally matches the data declared by the manufacturers (30–70 nm for ZrO_2_ and 150–200 nm for TiO_2_). It is also observed that, compared to the original ZrO_2_, the crystallites in the ceramics increase to submicron sizes due to the recrystallization effect upon heating, which is in general agreement with the results of the X-ray diffraction analysis. SEM analysis of the ceramic images shows the presence of large crystallites on their surface, more than 500 nm in size, which slightly diverges from the XRD results, according to which the average grain size in ceramics with an introduced TiO_2_ concentration of 5% is 150 nm. This result can be explained by the fact that the Scherrer method, used to determine the crystallite size from XRD data, does not reliably register particles larger than 500 nm, leading to somewhat underestimated grain size values.

**Figure 4:**
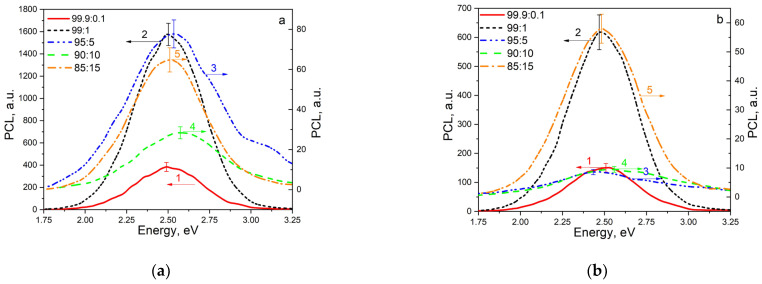
PCL spectra of various samples of unirradiated ZrO_2_:Ti ceramics (**a**) and ceramics irradiated with xenon ions with a fluence of 10^10^ ions/cm^2^ (**b**).

Figure 4 shows the PCL spectra of unirradiated ZrO_2_:Ti ceramics with different dopant concentrations, as well as samples exposed to xenon ion irradiation at a fluence of 10^10^ ions/cm^2^. All spectra contain a single broad band with a maximum at 2.5 eV. In this case, irradiation with xenon ions does not lead to the appearance of new PCL bands. Conversely, in ion-irradiated samples, a decrease in PCL intensity is observed compared to unirradiated samples with the same dopant concentration. This decline in luminescence intensity can be attributed to the destruction by irradiation-induced destruction of the luminescence centers responsible for emitting light at 2.5 eV, leading to the formation of more complex defects. The absence of any new luminescence bands related to the complex defects in the PCL spectra of irradiated ceramics (as shown in Figure 4b) can be explained by several factors. Firstly, it is plausible that these defects may emit light within a spectral range undetected by the PCL spectrometer, specifically in the UV range. Secondly, the low luminescence intensity could be due to the high likelihood of emission-free transitions occurring.

Figure 4 shows that the dependence of the maximum luminescence intensity in the 2.5 eV band on titanium concentration is non-monotonic. The highest PCL intensity in this band is characterized by samples with a concentration of introduced titanium dioxide of 1%. For ceramics containing higher concentrations of introduced titanium dioxide (5 and 10%), the intensity of this band notably decreases. At a titanium dioxide concentration of 15%, a slight increase in PCL intensity is noted at 2.5 eV.

**Figure 5:**
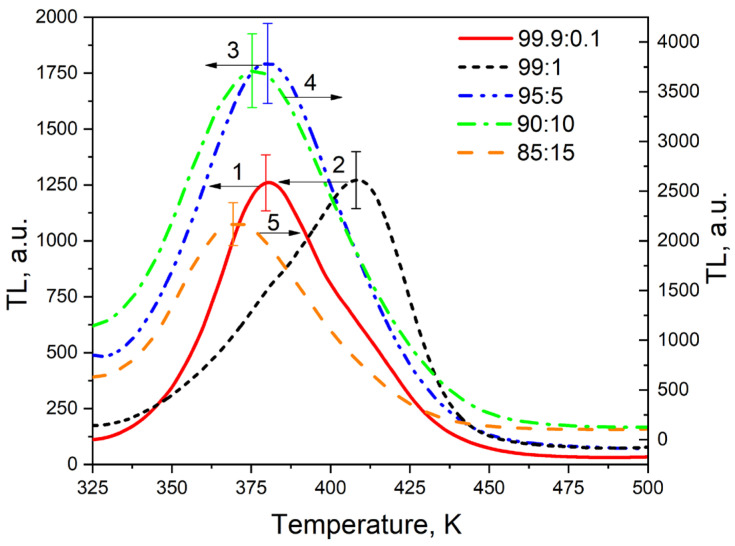
TL glow curves of samples with a concentration of introduced titanium dioxide of 0.1% (1), 1% (2), 5% (3), 10% (4), and 15% (5), irradiated with electrons at a dose of 15 kGy.

Figure 5 shows TL curves of ZrO2:Ti ceramics irradiated with a pulsed electron beam with a dose of 15 kGy, corresponding to concentrations of introduced titanium dioxide at 0.1%, 1%, 5%, 10%, and 15%.

For samples irradiated with electrons, an intense TL signal is observed at 325–450 K. The maximum intensity of thermoluminescence changes in a non-monotonic way with respect to the dopant concentration. Initially it increases, reaching a peak at 10% TiO_2_ content, before subsequently decreasing. Additionally, it is evident from the figure that both the position of the peak maximum and the shape of the TL curve vary with changes in the concentration of TiO_2_, indicating the complex structure of the traps responsible for the TL at 325–450 K.

We have decomposed the TL curves of Figure 5 to elementary peaks described by general-order kinetics. The following formulae were used for the decomposition of TL curves [53]:

(1) $$I\left(T\right)={\;I}_{m}b^{\frac{b}{b-1}}\exp\left(\frac{E}{\mathrm{kT}}\frac{T-T_{m}}{T_{m}}\right)\cdot {\left[\left(b-1\right)\left(1-\Delta \right)\frac{T^{2}}{T_{m}^{2}}\exp\left(\frac{E}{\mathrm{kT}}\frac{T-T_{m}}{T_{m}}\right)+Z_{m}\right]}^{-\frac{b}{b-1}}$$

(2) $$\Delta =2\mathrm{kT}/E,\;\Delta_{m}=2{\mathrm{kT}}_{m}/E,{\;Z}_{m}=1+\left(b-1\right)\Delta_{m},$$

where the variables represent the following.

I(T)—the TL intensity,

T (K)—the temperature,

k—the Boltzmann constant,

b—the order of kinetics,

E(eV)—the activation energy,

T_im_ (K)—the maximum temperature,

I_m_—the maximum intensity.

The values of b, E, T_m_, and I_m_ were variable parameters of Equation (1). The frequency factor was calculated using the formula:

(3) $$S=\frac{\beta E}{{\mathrm{kT}}_{m}^{2}}\frac{1}{Z_{m}}\exp\left(\frac{E}{{\mathrm{kT}}_{m}}\right)$$

The error of decomposition of TL curves into elementary peaks was estimated by the value of the FOM-factor [54] according to the formula:

(4) $$\mathrm{FOM}=\frac{\sum_{i}\left|I_{\exp}\left(T_{i}\right)-I_{\mathrm{fit}}\left(T_{i}\right)\right|}{\sum_{i}I_{\exp}\left(T_{i}\right)}\cdot 100\%,$$

where the variables represent the following.

I_exp_ (T_i_)—the set of TL curve intensity values obtained experimentally,

I_fit_ (T_i_)—the set of TL curve intensity values obtained theoretically by Formula (1).

**Figure 6:**
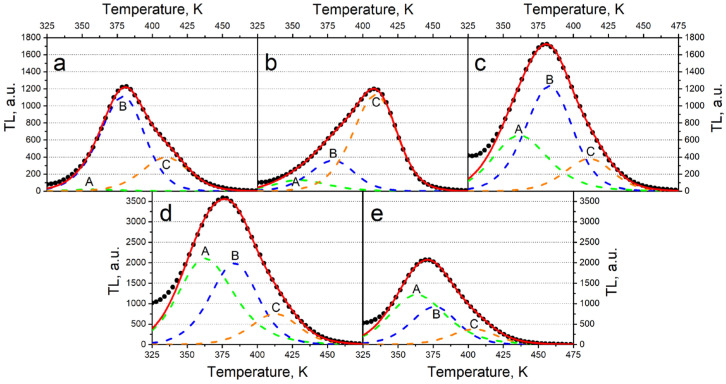
Results of the decomposition of TL curves into elementary peaks of samples with dopant concentrations of 0.1% (**a**), 1% (**b**), 5% (**c**), 10% (**d**), and 15%, (**e**) irradiated with electrons.

The results of decomposition of TL curves are shown in Table 2 and Figure 6. It is evident that all curves can be described as a combination of three basic peaks, A, B, and C. The calculated FOM values did not exceed 5%, which indicates a high degree of accuracy in the approximation performed [54]. At the same time, the values of the TL kinetic parameters of the indicated peaks (activation energy E, frequency factor S, and kinetic order b) vary only slightly from sample to sample, indicating the same nature of the traps. The TL activation energies of the A–C peaks generally align closely with values reported in [55] for the TL peak at 400 K in nominally pure samples of monoclinic ZrO_2_. However, unlike findings in [54], our TL curves demonstrate a non-elementary nature, indicating that titanium doping complicates the structure of TL curves in monoclinic zirconium dioxide ceramics.

**Figure 7:**
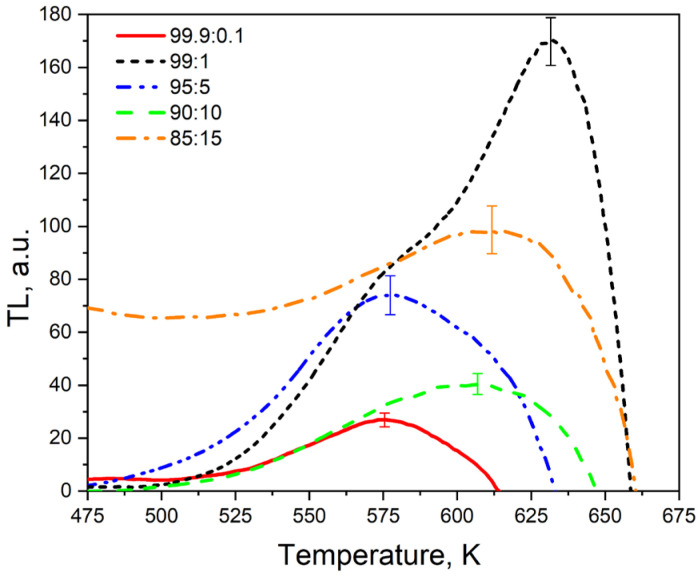
TL glow curves of samples with different concentrations of introduced titanium dioxide, irradiated with xenon ions with a fluence of 10^10^ ion/cm^2^.

The TL curves of the same samples irradiated with xenon ions exhibit significant differences from those of electron-irradiated samples (Figure 7). The low-temperature TL signal at 325–450 K is characterized by extremely low intensity, and a new high-temperature TL signal appears at 450–650 K. This signal may be attributed to the formation of new radiation-induced defects resulting from ion irradiation, which serve as traps for charge carriers.

The possibility of creating such defects was corroborated in [56] based on the analysis of the EPR spectra. Before irradiation, the EPR spectra of ZrO_2_ samples showed a signal from Zr^3+^ ions with a width of H_pp_ = 35 Gs and g = 1.963. However, after the samples were irradiated with a xenon ion beam (220 MeV), the EPR spectra changed significantly and no signal from Zr^3+^ ions was detected. Signals appeared at 3568 Gs (g = 1.963), 3500 Gs (g = 1.998), and 3525 Gs (g = 1.986). The signal with g = 1.998 (H_pp_ = 12 Gs) was attributed to the presence of F^+^ centers in the ion-irradiated samples. Signals with g = 1.986 and 1.963 (H_pp_ = 15 Gs) are attributed to a new previously unidentified radiation-induced center. The identical behavior of the signal intensities at g = 1.986 and 1.963 with changing the annealing temperature of the samples showed [56] that these signals belong to one paramagnetic center. The nature of this center is likely related to a complex defect, hypothesized to include paramagnetic Zr^3+^ ions and oxygen vacancies.

The low TL intensity observed at 300–450 K in ion-irradiated samples may be linked to variations in the mechanisms governing TL response formation in samples irradiated with electrons (130 keV) and ions (220 MeV). Electron irradiation at low energies (less than 1 MeV for zirconium dioxide [45,46]) primarily involves the occupation of traps and alterations in their charge states. In contrast, heavy ion irradiation may induce the creation of new defects within the material. Specifically, ion irradiation can result in the radiation-induced damage of traps responsible for the TL signal at 325–450 K and the formation of new trapping centers, which become depleted at 450–650 K.

Similar to the patterns observed in PCL and TL signals within the 325–450 K range, the dependence of TL intensity at 450–650 K on titanium concentration is non-monotonic. The highest intensity is observed for an introduced titanium dioxide concentration of 1%, which coincides with the findings from the analysis of PCL spectra. A further increase in dopant concentration causes a decrease in TL intensity. Conversely, at an introduced titanium dioxide concentration of 15%, an increase in the TL signal is observed, mirroring the observations in PCL (Figure 4).

A non-monotonic dependence of both PL and PCL intensity on the concentration of titanium for monoclinic ZrO_2_ samples not subjected to any irradiation was observed in [47]. Moreover, the decrease at a high dopant content was associated with concentration quenching of the luminescence. It is plausible that concentration quenching has also affected our samples. This phenomenon may occur when sufficiently high concentrations of dopant disrupt the mutual isolation of the luminescence centers. Consequently, the interaction among these centers reduces the probability of the radiative transitions. This interaction can involve resonant energy transfer from one impurity ion to another, continuing until this energy is intercepted by a quencher. Such a process is most probable when the excited state has a long lifetime. A cross-relaxation interaction between luminescence centers can decrease yield with increased dopant concentration [57]. In [58], an interpretation of the concentration quenching effect based on the analysis of TL kinetics was proposed. The model’s zone scheme includes three interacting electron traps and one recombination center. The variable parameter was the total concentration of luminescence centers (M), with the initial occupancy of luminescence centers by holes not assumed to be zero. It is shown that the non-monotonic change in TL yield of two low-temperature peaks with the variation in M is due to the competitive interaction between traps during excitation and heating of the sample.

**Figure 8:**
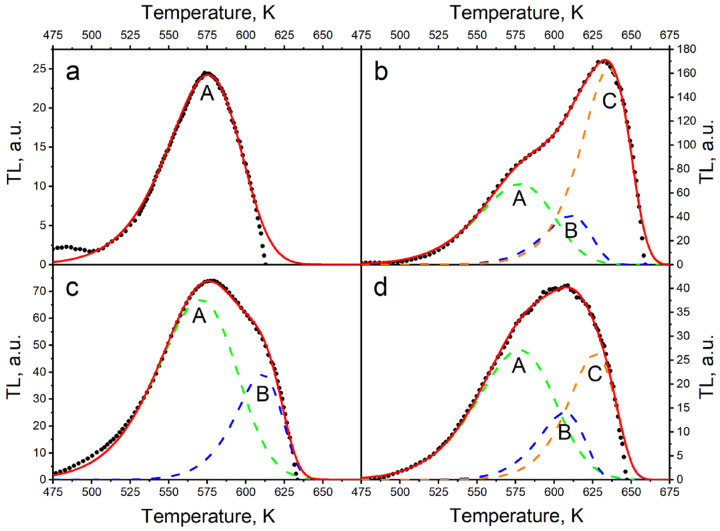
The results of decomposition into elementary peaks of the TL curves of samples with a concentration of introduced titanium dioxide of 0.1% (**a**), 1% (**b**), 5% (**c**), and 10% (**d**).

Further study is required to interpret the increase in the intensity of PCL and TL signals at 450–650 K at the maximum concentration of TiO_2_ (15%). It can be assumed that at a high concentration of dopant the probability of tunnel transitions between traps and luminescence centers increases. These transitions create an additional radiative relaxation channel, which can increase luminescence intensity. In favor of a possible role of tunneling in the formation of luminescence of the studied samples, the presence of an extended temperature-independent horizontal part on the TL curve at T = 475–550 K of ceramics with a maximum concentration of titanium (15%) irradiated with xenon ions can testify (Figure 7). It is known that the tunneling recombination mechanism is in principle a temperature-independent process [59]. Figure 7 shows that the TL curves of the ion-irradiated samples have a complex shape, which is a superposition of several TL peaks. This is further supported by the shift in the TL maximum temperature with variations in dopant concentration. To determine the TL kinetic parameters of the investigated samples, the TL curves were decomposed into elementary peaks described by general order kinetics. The curve decomposition was not carried out for samples having the maximum concentration of introduced titanium dioxide (15%) since the TL curve at T = 475–550 K has a shape resembling a plateau. This indicates the existence of tunneling processes or a continuous energy distribution of traps that are responsible for thermoluminescence. It is well-established that an excessive concentration of dopant in a material can lead to a significant disorder in its structure. This can result in noticeable changes in the crystal lattice parameters (as shown in Table 1), and an increase in the complexity of the energy spectrum of the capture centers [53]. Formulas (1)–(4) were used to decompose the TL curves, and the results are presented in Table 3 and Figure 8. The data from Table 3 indicate that samples with the lowest dopant concentration (0.1%) have a TL curve that can be approximated by a single elementary peak (Figure 8a). For samples with a 5% concentration of titanium dioxide, the TL curve is a superposition of two peaks A and B (Figure 8c). The TL curves for the remaining samples were described by the sum of at least three peaks (A–C), as shown in Figure 8b,d. The calculated FOM values did not exceed 5%, indicating the high accuracy of the performed approximation [53]. An important finding from Table 3 is that the values of the A–C peak parameters (E, S, and b) are similar for samples with varying dopant concentrations. This suggests the general nature of radiation-induced traps responsible for the TL signal at 475–650 K in ion-irradiated samples. The close to unity order values of the TL kinetics indicate an insignificant role of the processes of carrier recapture in the traps during thermo-stimulation.

## 4. Conclusions

In this study, zirconium dioxide ceramics with varying concentrations of titanium impurity ions were synthesized. According to X-ray diffraction measurements, all samples contain 100% monoclinic phase. The observed shifts of the diffraction reflection maxima are caused by distortions of the crystal lattice of ZrO_2_ during doping. It was found that the irradiation of samples with high-energy xenon ions resulted in a reduction in the PCL intensity at 2.5 eV. In this case, no new emission bands were detected in the PCL spectra. The decrease in luminescence intensity could potentially be due to the destruction of the luminescence centers responsible for the formation of the 2.5 eV band by irradiation. Based on the findings of this work, it is not possible to precisely determine the nature of the luminescence centers at 2.5 eV in the studied ceramics. It can only be asserted that titanium ions participate in their formation. This study demonstrates that ion irradiation, unlike electron irradiation, results in the emergence of a new TL signal at 450–650 K, presumably associated with radiation-induced defects, which are traps of charge carriers. Through the decomposition into elementary peaks, the values of the kinetic parameters of TL are calculated. A complex non-monotonic dependence of both the PCL and TL intensity on the concentration of titanium impurity was found, potentially attributed to concentration quenching and the presence of the tunneling transitions of charge carriers.

## Figures and Tables

**Table 1:** Results of X-ray phase analysis of ZrO_2_:Ti samples.

Sample (ZrO_2_:TiO_2_)	Lattice Parameters, Å	Reflex 2θ = 28.3° Half-Width	Crystallite Size, nm
99.9:0.1	a = 5.1493 b = 5.2013 c = 5.32003 beta = 99.23°	0.1514	200
99:1	a = 5.1464 b = 5.1996 c = 5.3178 beta = 99.22°	0.1554	180
95:5	a = 5.1360 b = 5.1801 c = 5.3167 beta = 99.15°	0.1634	150
90:10	a = 5.1265 b = 5.1535 c = 5.3216 beta = 99.32°	0.2070	85
85:15	a = 5.1115 Å b = 5.1344 Å c = 5.3223 Å beta = 98.82°	0.2121	80

**Table 2:** Results of the decomposition of TL curves of electron-irradiated samples into elementary peaks.

Peak	Parameter	Impurity Level (ZrO_2_:TiO_2_)				
		99.9:0.1	99:1	95:5	90:10	85:15
A	T_m_, K	358	355	361	363	363
	E, eV	0.80	0.80	0.80	0.80	0.80
	S, s^−1^	2.5·10^10^	3.1·10^10^	1.9·10^10^	1.7·10^10^	1.7·10^10^
	b	2.00	1.96	2.00	2.00	2.00
B	T_m_, K	379	379	383	384	376
	E, eV	0.94	0.95	0.94	0.93	0.94
	S, s^−1^	5.1·10^11^	5.7·10^11^	3.1·10^11^	2.3·10^11^	5.5·10^11^
	b	1.51	1.51	1.59	1.58	1.61
C	T_m_, K	410	409	411	413	404
	E, eV	1.09	1.11	1.08	1.09	1.10
	S, s^−1^	3.2·10^12^	6.8·10^12^	2.7·10^12^	3.0·10^12^	8.2·10^12^
	b	1.58	1.49	1.59	1.60	1.59
FOM, %		2.7	2.4	4.4	4.5	3.4

**Table 3:** Decomposition of TL curves of the samples irradiated with xenon ions to elementary peaks.

Peak	Parameter	Impurity Level (ZrO_2_:TiO_2_)			
		99.9:0.1	99:1	95:5	90:10
A	T_m_, K	575	577	570	577
	E, eV	1.23	1.23	1.14	1.20
	S, s^−1^	5.2·10^9^	4.7·10^9^	9.7·10^8^	2.4·10^9^
	b	1.11	1.14	1.17	1.19
B	T_m_, K	-	611	611	607
	E, eV	-	2.09	2.11	2.10
	S, s^−1^	-	2.5·10^16^	3.2·10^16^	3.4·10^16^
	b	-	1.00	1.00	1.00
C	T_m_, K	-	636	-	627
	E, eV	-	2.20	-	2.20
	S, s^−1^	-	3.4·10^16^	-	6.1·10^16^
	b	-	1.00	-	1.00
FOM, %		2.9	4.6	2.9	3.3

## Data Availability

The data sets are available from the corresponding author upon reasonable request.
